# The effect of Covid-19 pandemic on healthcare utilization in public vs private centers in Iran: a multiple group interrupted time-series analysis

**DOI:** 10.1186/s12913-023-09846-1

**Published:** 2023-08-01

**Authors:** Zohreh Rezaei, Farhad Lotfi, Mohsen Bayati, Zahra Kavosi

**Affiliations:** 1 Student Research Committee, School of Health Management and Information Sciences, Shiraz University of Medical Sciences, Shiraz, Iran; 2 Health Human Resources Research Center, School of Health Management and Information Sciences, Shiraz University of Medical Sciences, Almas Building, Alley 29, Qasrodasht Ave, Shiraz, 71336-54361 Iran

**Keywords:** Covid-19, Utilization, Time-Series Analysis, Access, Pandemic, Healthcare

## Abstract

**Background:**

The outbreak of Coronavirus in late 2019 and its continuation in the following years has affected all human societies, government organizations, and health systems. Access to health services is an important issue during crises. The present study aimed to investigate the effect of the Covid-19 pandemic on the consumption of health services in the public sector compared to the private sector in Iran.

**Methods:**

The research population consisted of all insured individuals covered by Iran Health Insurance Organization in Fars province, which amounts to approximately 2,700,000 people. The required information including the utilization of laboratory, radiology, medicine, and hospitalization services was extracted on a monthly basis from February 2019 to February 2021. The Multiple Group Interrupted Time Series Analysis (MGITSA) was used for data analysis along with STATA.15 software.

**Results:**

According to the findings of MGITSA, in the short-term, the utilization of private laboratory, radiology, medication, and hospital admissions had decreased by approximately 18,066, 8210, 135,445, and 1086 times, respectively (*P* < 0.05). In the long-run, the use of laboratory and radiology services had increased by about 2312 and 514 times (*P* < 0.05), respectively. The comparison between the public and private sectors showed that in the short-term, the use of radiology services decreased by about 12,525, while the use of medication increased by about 91,471 times (*P* < 0.05). In the long-run, the use of laboratory services decreased by about 1514 times (*P* = 0.076) and no change was observed in the other services utilization (in public relative to private centers).

**Conclusions:**

Utilization of health services in the public versus private centers, except for medication and hospitalization, significantly decreased in the short-term. However the utilization of most services returned to the usual trend in the long-term. The reduction in access to health services could impose a significant burden of various diseases, at least in the short-term, and increase health costs in the coming years.

## Background

The outbreak of Covid-19 in late 2019 and its continuation in the following years has affected all human societies, government organizations, and health systems [1], and it is currently one of the most important health issues [2]. Since the outbreak of the corona virus and its spread to almost all countries (over 211 countries so far), efforts have been made to plan for prevention and control the disease [3]. According to the latest World Health Organization (WHO) reports, about 255 million cases and 5 million deaths were recorded until November 2021, and this statistic is increasing daily [4].

The high rate and prevalence of the Covid-19 virus have caused the disease to have many effects on social factors and consumer behavior [5]. Additionally, the prevalence of Covid-19 is one of the factors affecting access to health services [6]. Access to health services has been an important issue in health policy-making and has been defined as the coupling of the supply and demand sides of health and medical care [3, 7]. Since direct measurement of access to health services is practically impossible, demand or utilization is used to measure it.

The WHO has reported that the Covid-19 pandemic has had a significant impact on the provision or utilization of non-communicable disease healthcare, resulting in partial or complete disruption of health services in many countries. In its report, the WHO stated that in more than half (53%) of the countries surveyed, hypertension treatment services were partially or completely disrupted. The percentage was estimated at 49% for the treatment of diabetes and its complications, 42% for cancer treatment, 31% for cardiovascular emergencies, and 63% for rehabilitation services [8]. In Korea general population, it was reported that due to pandemic, the utilization of many health services such as health screenings (25.8%), non-urgent medical visits (7.6%), medical visits for chronic disease (10%), and emergency visits (39.6) has been delayed [9].

The outbreak of Covid-19 has reduced access to health services, including surgeries, elective procedures, and emergency services. Concerning emergency services, this is particularly important as it may impact people's health and also increase health system costs and disease burden in the coming years. A recently published systematic review on Coivid-19 pandemic effects on the patients with Type 2 Diabetes Mellitus indicated the decrease in routine healthcare utilization, delay in service delivery, and noticeable increase in telemedicine utilization [10].

Data from the USA, Italy, and Spain showed a reduction of at least 50% in hospital admissions for myocardial infarction. This data indicates that patients who experienced chest pain or myocardial infarction symptoms during the Covid-19 pandemic were less likely to call the emergency room or refer to the hospital [11]. Some studies also reported a significant reduction in hospitalization (during COVID-19 outbreak) of the patients with various acute medical conditions such as heart failure and acute coronary syndrome [12–14]. In china it was shown a decrease in healthcare utilization such as outpatient services, prenatal care visits, chronic care and emergency department visits. The greatest reduction was reported for preventive care visits. After reopening period, visits of mental and sleep disorders, and medical abortion increased [15].

In Iran, primary health care is delivered by public sector and has always been a state obligation. Secondary and tertiary level healthcare are provided by both public and private sectors. Recent statistics show almost 84% of inpatient services (117 000 hospital beds equals 1.62 per 1000 population) bed and more than 80% of outpatient services are provided by public and private sectors respectively [16].

As of writing this article, no published original study has been found on the effects of Covid-19 on healthcare services utilization in Iran. Only in one letter, a reduction of about 56% in neurosurgeries at Rasoul Akram Hospital in Tehran was reported [17]. According to studies, several factors affect the utilization of medical services, including gender, age, education level, occupation and income, type of insurance, doctors' advice, distance to healthcare facilities, and quality of services [18–20].

The present study aimed to investigate the effect of the Covid-19 pandemic on the utilization of various health services. Due to the different characteristics of the public and private sectors, researchers assumed that the pandemic would have different effects on the use of services in these two sectors. In the public sector, there was a higher likelihood of infection due to the contamination of public facilities with the virus. On the other hand, emergency services were mainly provided by the public sector, and their utilization might be less affected by the outbreak of the disease. Conversely, in the private sector, there was less concern about Covid-19, and the services provided were mainly elective and non-essential. Despite the importance of this issue, there has been very little comprehensive and detailed study to compare the impact of the pandemic on the consumption of healthcare utilization in the private and public sectors, especially in developing countries. Therefore, more precisely, the present study aimed to investigate the impact of the Covid-19 pandemic on the utilization of various health services in the public sector compared to the private sector.

## Methods

This study was conducted as a time series within a 24-month period (before and during the Covid-19 pandemic) in Fars province which has an area of approximately 125,000 square kilometers. According to the latest census of the Statistical Center of Iran, Fars was the fourth most populous province in the country in 2016, with a population of 4,851,274 [21]. Having 60 public, university, and private hospitals and educational and medical centers, as well as 6,662 hospital beds in Shiraz and other cities, it is known as the center of medical services in the southern of Iran [22].

The present study was conducted on insured population covered by Iran Health Insurance Organization in Fars province (n ≈ 2,700,000 of people equals to 55% of population) in 2021. Given that the general level of service volume was used for this study, the whole population was surveyed and no sampling was carried out.

The data on the monthly utilization of health services was extracted from February 2019 to February 2021 (12 months before and 12 months after the onset of the pandemic in Iran) from the insurance information system of the Fars Health Insurance Organization, and were classified by public and private centers. The studied variables included the frequencies of laboratory tests, radiology services, hospitalization, and drug consumption. February 2020 (the onset of the pandemic in Iran) was considered as interrupted time. The short-term (1-month) and long-term (12-month) effects of the pandemic on each of the utilization variables were then investigated.

To analyze the data, descriptive statistics and univariate analysis (using the Mann–Whitney test) were first applied to compare the average number of utilization one year before and one year after the onset of Covid-19.

The use of routine epidemiologic methods such as randomized clinical trials to investigate the impact of various interventions has many limitations due to ethical or political reasons, the impossibility of randomization, and the need to evaluate a retrospective intervention. Especially when there are interventions or events at the society level, it is practically impossible to use those methods. One of the alternative methods as a quasi-experimental designs is the Interrupted Time Series Analysis (ITSA) which is increasingly being used. This is particularly useful for evaluating the impact of public health interventions or events that affect the health outcomes. It is especially used for interventions or events in a certain time period and determining its health outcomes impact at the population level [23, 24].

ITSA has been used for the assessment of a different type of interventions or event including health program or reforms, vaccination, lockdown, legislation for helmet use, traffic speed zones, and so on. It was also used for the impact evaluation of unintentional events such as Covid-19 pandemic [23, 25–28].

In general model of ITSA, the two variables including level and trend variable are used to indicate the impact of an intervention/event. The immediate and long-term effects of the interventions, are derived from level and trend variable, respectively. The following regression model shows a simple ITSA [29].

$${\mathrm{Y}}_{\mathrm{t}}={\upbeta }_{0}+{\upbeta }_{1}{\mathrm{T}}_{\mathrm{t}}+{\upbeta }_{2}{\mathrm{X}}_{\mathrm{t}}+{\upbeta }_{3}{\mathrm{T}}_{\mathrm{t}}{\mathrm{X}}_{\mathrm{t}}+{\mathrm{E}}_{\mathrm{t}}$$

where Y_t_, T, and X_t_, represent the outcome variable, time trend, and intervention/event, respectively. T_t_X_t_ is the interactive effects of time and intervention, and e_t_ indicates the error term of model. The X_t_, is a dummy variable equals to 1 for after the intervention points and 0 for before that). In the model, β0, β1, β2 and β3 show the intercept or the outcome level before the intervention, time trend before the intervention, changes in outcome level after intervention (immediate effect), and changes in outcome trend after intervention (long-term effect), respectively [29].

When the intervention is performed in more than one group and the effect of the intervention is different in different groups, the Multiple Group ITSA (MGITSA) should be used. Capability to assess comparability between groups on covariates is a main strength of this analysis [29].

In the current study the MGITSA method was then used to investigate the short-term and long-term effects of the pandemic on the use of health services in the public sector compared to the private sector. The following segmented regression was also used to analyze the model.

$${\mathrm{Healthcare \ Utilization}}_{\mathrm{t}}={\upbeta }_{0}+{\upbeta }_{1}{\mathrm{T}}_{\mathrm{t}}+{\upbeta }_{2}{\mathrm{X}}_{\mathrm{t}}+{\upbeta }_{3}{\mathrm{T}}_{\mathrm{t}}{\mathrm{X}}_{\mathrm{t}}+{\upbeta }_{4}\mathrm{Z}+{\upbeta }_{5}{\mathrm{ZT}}_{\mathrm{t}}+{\upbeta }_{6}{\mathrm{ZX}}_{\mathrm{t}}+{\upbeta }_{7}{\mathrm{ZT}}_{\mathrm{t}}{\mathrm{X}}_{\mathrm{t}}+{\mathrm{e}}_{\mathrm{t}}$$

Yt: shows healthcare utilization including the laboratory, radiology, hospital admission and medication. Tt: the time since the start of the study, a continuous variable. Xt: is a dummy variable representing the prevalence of the pandemic, (pre-pandemic periods 0, post-pandemic periods 1). Z: is a dummy variable representing the groups, (private health centers 0, and public health centers 1). TtXt, ZTt, ZXt and ZTtXt: are interaction terms among Z, Tt and Xt. β0: represents the intercept or starting level of the healthcare utilization for private health centers. β1: is the slope or trend of the healthcare utilization until initiation of the pandemic for private health centers. β2: represents the change in the level of the healthcare utilization that occurs immediately following initiation of the pandemic (for private health centers). β3: represents the difference between pre-pandemic and post- pandemic slopes of the healthcare utilization (for private health centers). β4: represents the difference in the level of the healthcare utilization between public and private health centers prior to the pandemic. β5: represents the difference in the trends of the healthcare utilization between public and private health centers prior to the pandemic. β6: represents the difference between public and private health centers in the level of the healthcare utilization immediately following initiation of the pandemic. β7: shows the difference between public and private health centers in the slope after the pandemic compared with pre-pandemic. et: is the error term of model.

For analysis, all of the data were first entered the Excel software and were aggregated. Then the “itsa” Package of STATA.15 was used to analyze the data and draw the graphs and tables. The effect of an intervention or event on a time series outcome is estimated by itsa package. In itsa, coefficients Newey-West standard errors is estimated by OLS regression. It is assumed the errors in estimated linear regression model follow first-order autoregressive. This package can used for estimation of treatment effects for either single or multiple groups comparison [29].

This study was approved by the ethics committee of the Shiraz University of Medical Sciences.

## Results

The results of the mean difference test showed that after the Covid-19 pandemic, there was an insignificant decrease in the utilization of laboratory services and hospitalization in the private sector. However, there was a significant decrease in the utilization of laboratory services and hospitalization in the public sector after the Covid-19 pandemic. Additionally, both public and private sectors showed a significant decrease in the consumption of radiology services and medication (Table 1).

**Table 1:** Difference between average monthly utilization of health services in public and private sectors before and after Covid-19 pandemic in Fars province

**Services**		**Pre mean (Standard deviation)**	**Post mean (Standard deviation)**	**Z**	***P*-value**
**Laboratory**	**Private**	29,853.7 (2056.7)	26,966.3 (11,050.7)	1.414	0.157
	**Public**	45,019.5 (5887.1)	26,995.3 (6645.7)	4.134	< 0.001
	**Total**	74,873.3 (7411.8)	53,961.6 (15,622.4)	3.372	< 0.001
**Radiology**	**Private**	13,718.4 (1852)	9665.4 (3143.3)	3.427	< 0.001
	**Public**	33,703.5 (3744.9)	21,337.5 (5291.9)	4.134	< 0.001
	**Total**	47,422 (5478.8)	31,003 (8400.9)	3.807	< 0.001
**Medicine**	**Private**	293,188.1 (34,968.3)	207,189.4 (28,315.1)	4.025	< 0.001
	**Public**	70,244 (6709.7)	36,633.6 (5107.9)	4.243	< 0.001
	**Total**	363,432.1 (40,440.5)	243,823.1 (33,055.8)	4.243	< 0.001
**Hospital** **Admission**	**Private**	1871.3 (446)	1809.9 (573.9)	0.054	0.956
	**Public**	20,077.7 (1427.9)	18,548.3 (11,378.2)	2.937	0.003
	**Total**	21,949 (1760.7)	20,358.2 (11,100)	2.883	0.003

**Fig. 1:**
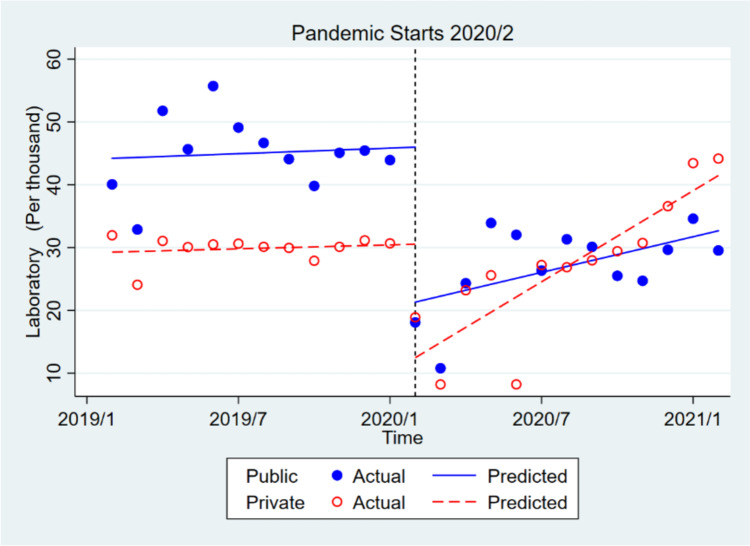
Effect of covid-19 pandemic on the laboratory services in public health centers compared to private health centers

In the private sector, the utilization of laboratory services decreased significantly by about 18,066 times in the short term, but it increased insignificantly by about 2312 times in the long run. The initial monthly use of laboratory services before the pandemic was about 14,924 times higher in the public sector than the private sector, which was significant. However, the trends of pre- pandemic consumption in the public and private sectors were not significantly different. The monthly utilization of laboratory services in the public sector, compared to the private one, decreased insignificantly by about 6596 times after the onset of the pandemic (short-term), and the consumption trend in that sector decreased significantly (at 10% level) by about 1514 times in the long run (Table 2, Fig. 1).

**Table 2:** Multiple group analysis of the effect of Covid-19 pandemic on the health services in public health centers compared to private health centers

			**Laboratory**		**Radiology**		**Medicine**		**Hospital Admission**	
			Coefficient	*P*-value	Coefficient	*P*-value	Coefficient	*P*-value	Coefficient	*P*-value
**Private health centers**	**Before Covid-19**	(β0) Mean value at the baseline	29,281	< 0.001	13,248.5	< 0.001	266,861	< 0.001	1464.1	< 0.001
		(β1) Pre-trend	104.1	0.645	85.4	0.600	4786.7	0.119	74	0.039
	**After Covid-19**	(β2) Post-level change	-18,066.7	< 0.001	-8210.4	< 0.001	-135,445.1	< 0.001	-1086.3	0.008
		(β3) Post-trend change	2312.9	< 0.001	514.9	0.038	-1731.3	0.669	16.5	0.747
**Public health centers** **relative to private health centers**	**Before Covid-19**	(β4) Pre-level difference	14,924.9	0.002	18,211.8	< 0.001	-203,025.3	< 0.001	17,639.1	< 0.001
		(β5) Pre-trend difference	43.8	0.937	322.4	0.359	-3621.5	0.239	103.1	0.397
	**After Covid-19**	(β6) Post-level difference	-6596	0.282	-12,525.8	0.001	91,471.2	0.002	6397	0.443
		(β7) Change in slope difference pre-to post	-1514.7	0.076	30.4	0.952	1031.1	0.801	-1525.7	0.108
	**Model** **significance**	F	27.84		104.70		575.95		736.65	
		*P*-value	< 0.001		< 0.001		< 0.001		< 0.001

**Fig. 2:**
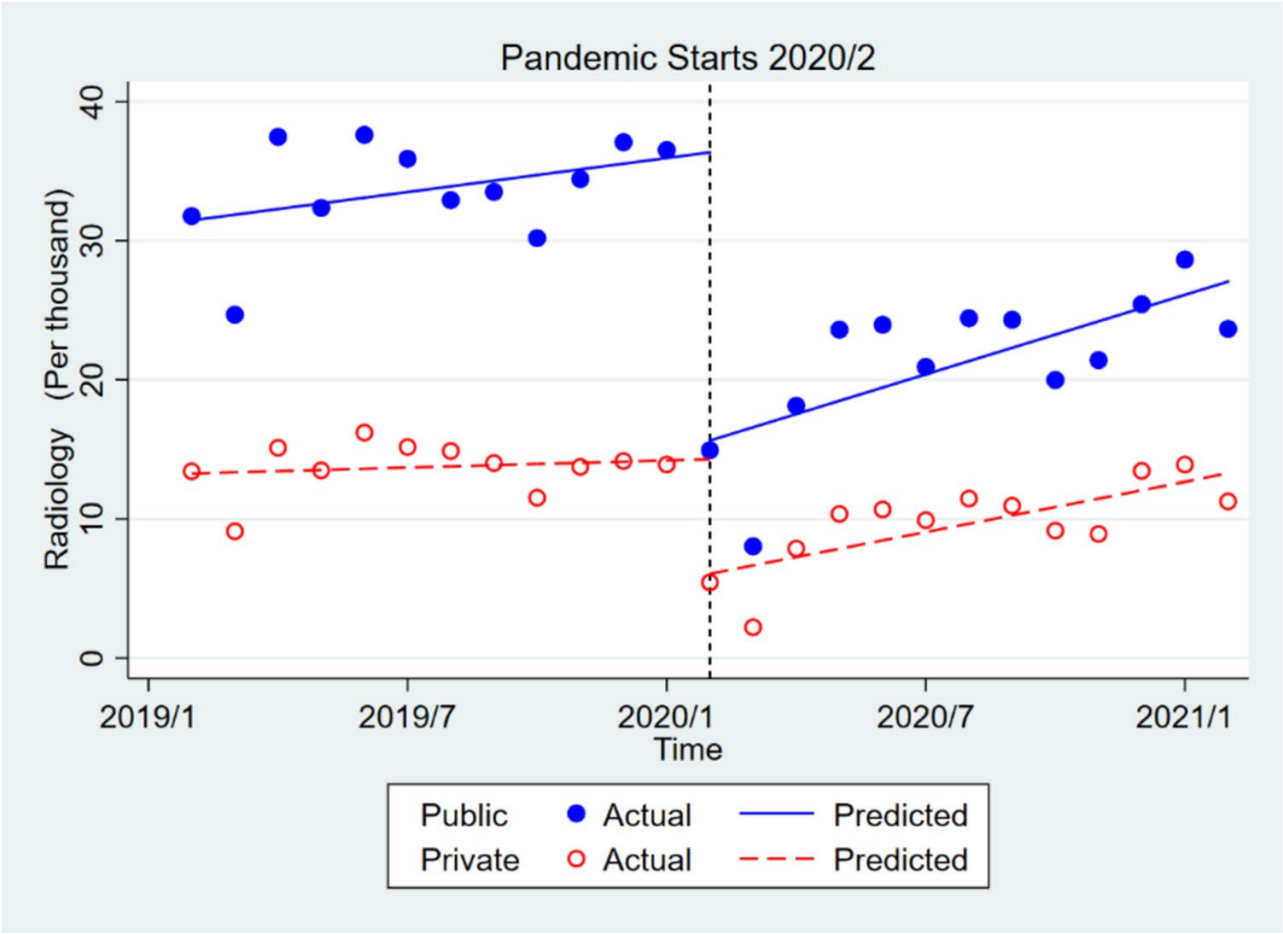
Effect of covid-19 pandemic on the radiology services in public health centers compared to private health centers

In the private sector, the utilization of radiology services decreased significantly by about 8210 times in the short term, and increased significantly by about 514 times in the long run. The initial monthly use of radiology services before the epidemic in the public sector was about 18,211 times higher than in the private sector, which was significant, and the trends of pre-pandemic consumption in the public and private sectors were not significantly different. The monthly consumption of radiology services in the public sector, compared to the private one, decreased significantly by about 12,525 times after the pandemic (short-term), and the trend of consumption in the public sector increased insignificantly by about 30 times in the long run (Table 2, Fig. 2).

**Fig. 3:**
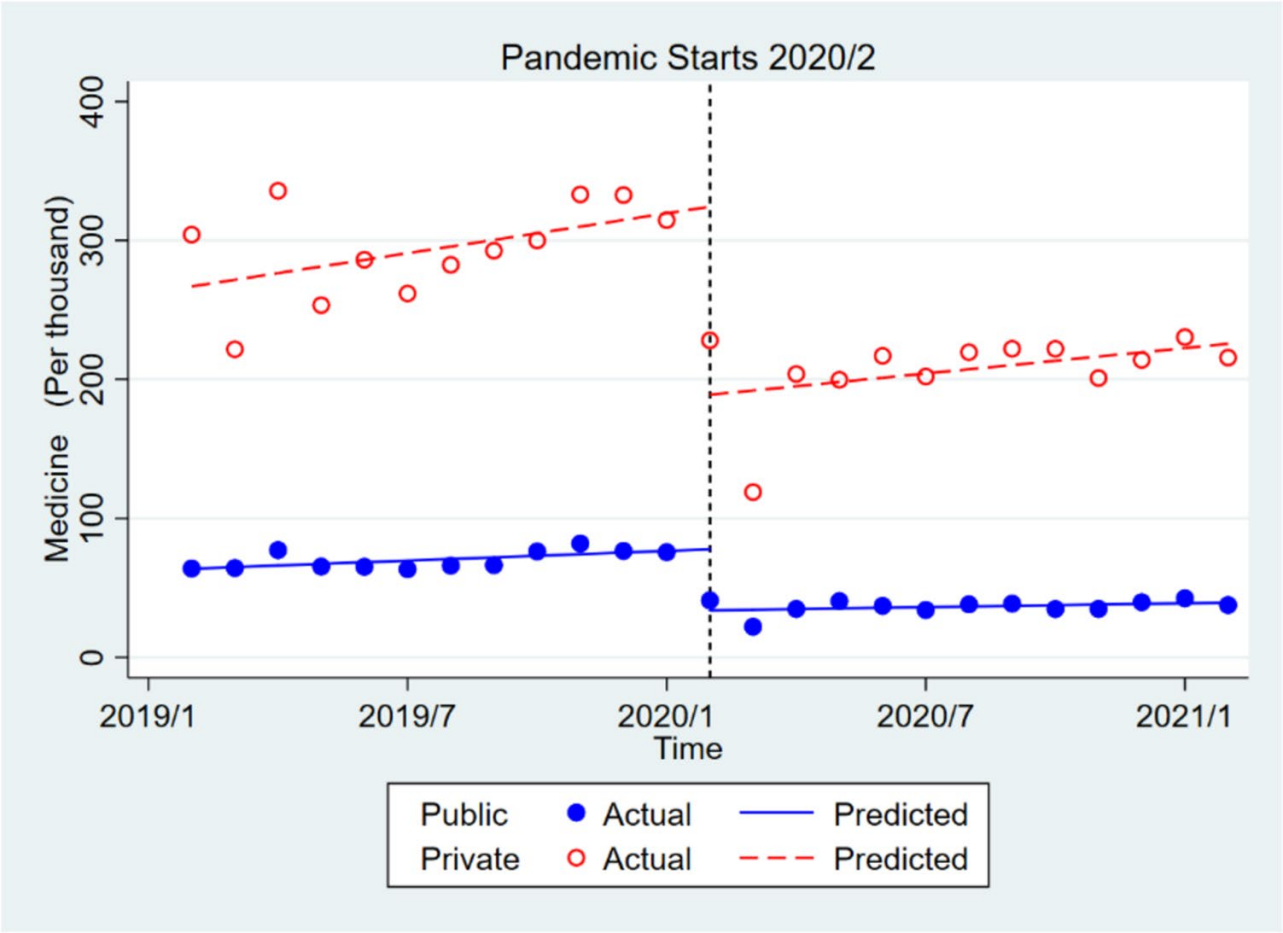
Effect of covid-19 pandemic on the medicine in public health centers compared to private health centers

In the private sector, drug consumption decreased significantly by approximately 135,445 times in the short term and insignificantly by about 1731 times in the long run. The initial monthly use of drugs before the pandemic in the public sector was about 203,035 times less than in the private sector, which was significant. However, the trends of pre- pandemic drug consumption in the public and private sectors were not significantly different. The monthly drug consumption in the public sector, compared to the private one, increased significantly by about 91,471 times after the pandemic (short-term), and the consumption trend in the public sector, compared to the private one, increased insignificantly by about 1031 times in the long run after the pandemic (Table 2, Fig. 3).

**Fig. 4:**
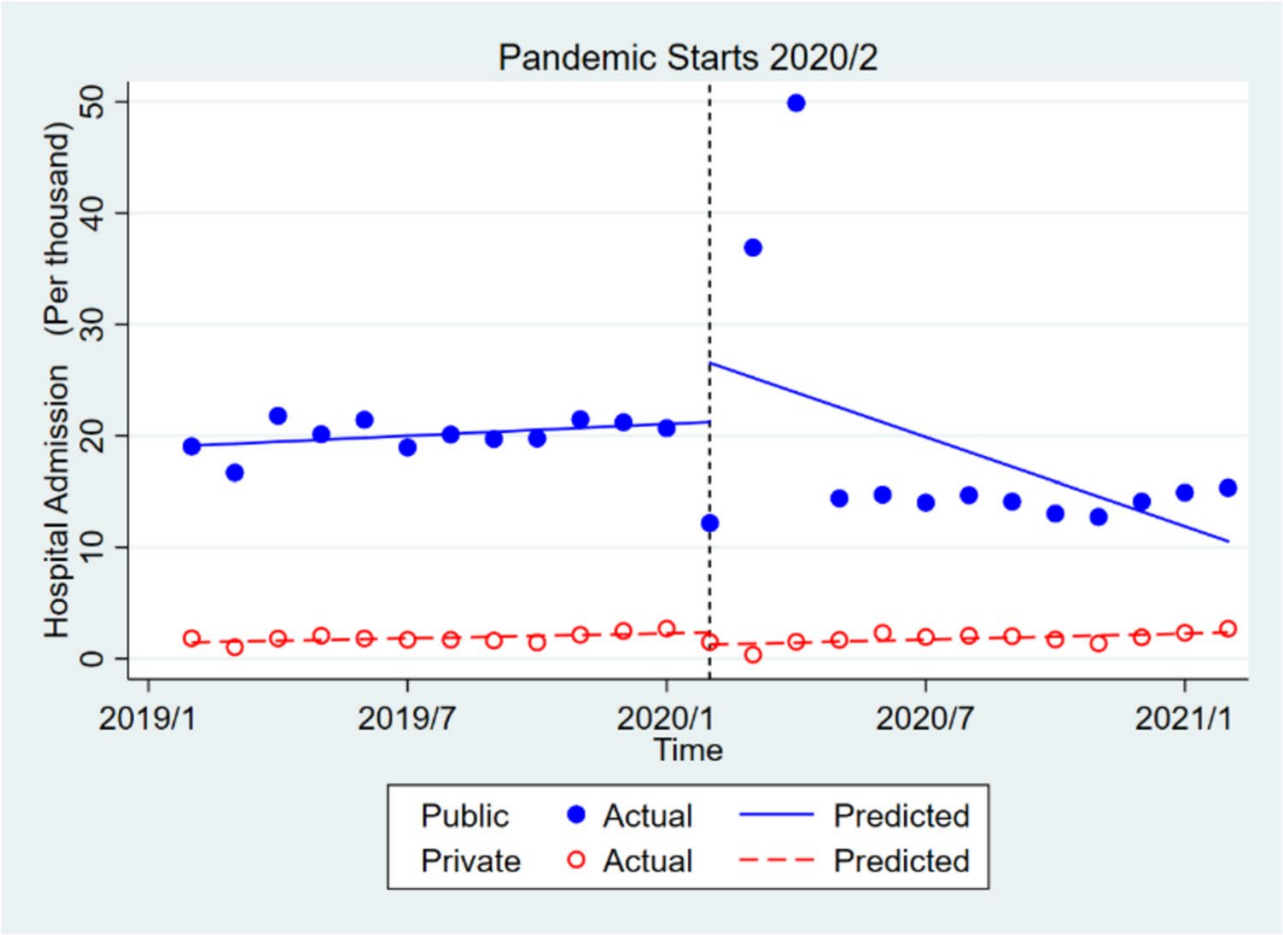
Effect of covid-19 pandemic on the hospital admission in public health centers compared to private health center

In the private sector, inpatient admissions decreased significantly by almost 1086 times in the short term but increased insignificantly by about 16 times in the long run. The initial monthly pre- pandemic inpatient admission in the public sector was about 17,639 times higher than in the private sector, which was significant. However, the trends of pre-pandemic hospital admissions in the public and private sectors were not significantly different. The monthly hospital admissions in the public sector increased insignificantly by about 6397 times compared to the private sector in the short run after the pandemic. However, in the long term the admission trend in the public sector (compared to the private one) decreased significantly by approximately 1525 times (Table 2, Fig. 4).

The significance of all models are confirmed based on the F-statistic (Table 2).

## Discussion

This study examined the impact of the Covid-19 pandemic on the utilization of health services in the public and private sectors, considering the likelihood of its effects on the utilization of health services and the future burden of various diseases. The findings of this study are discussed separately in terms of laboratory services, radiology, medicine, and inpatient services.

The findings showed that the Covid-19 pandemic reduced the utilization of private laboratory services in the short term and increased it in the long term. In the public sector, relative to private sector, however, the use of laboratory services did not change in the short term, but decreased in the long run. The reduced utilization of services in the short term could be attributed to closure policies and people's fear of contracting the virus [30, 31]. On the other hand, the increase in service utilization in the long run could be contributed to the prolonged duration of the pandemic and the people’s increased need to seek healthcare services. Furthermore, the decreased consumption of services in the public sector in the long run, compared to the private sector, could be attributed to the people’s preference for using cleaner and more secluded laboratories during the pandemic. In their study in the United States, Ziedan et al. showed that the average weekly utilization of non-Covid-19 health care services, including visits for chronic care procedures and laboratory tests such as cardiac stress testing, chemotherapy, cancer screening, diagnostic imaging, and other repeated blood tests, decreased from about 11,000 laboratory tests per week to about 9,000 during the early weeks of the lockdown. State closure policies were associated with a 25–30% reduction in total laboratory testing and were considered as a major factor in reducing the utilization of laboratory services in the country [31]. Nikolayevskyy et al. in Europe also reported that in the short term, the services provided by European National tuberculosis Reference Laboratories (NRLs) were affected by persistent restrictions, quarantines, staffing shortages, and logistics issues, leading to the suspension of certain services. Such changes are likely to have a significant long-term impact on the ability of NRLs to respond to current and future tuberculosis-related threats [30]. Yucel et al. in Turkey reported an average decrease of 68.3% in the number of patients and laboratory biochemical tests in April 2020 compared to April 2019. However, the number of Covid-19 diagnostic tests increased [32].

Based on the findings, the Covid-19 pandemic resulted in a reduction in the utilization of private radiology services in the short term, but an increase in the long term. In the public sector, compared to the private one, the utilization of radiology services decreased in the short term, but did not show any changes in the long term. The reduced consumption of services in the short term could also be attributed to closure policies and people's fear of contracting the virus [33–35]. It appears that the increased consumption of services in the long run could be attributed to the prolonged duration of the pandemic and people's increased need to seek healthcare service. In addition, the need for scans to detect lung involvement in this pandemic has led to an increased utilization of radiology services in the private sector in the long-term, possibly due to people's preference for cleaner and more secluded environments and their belief in the higher accuracy of private centers in Iran.

Nadich et al. in New York examined the weekly imaging volume and reported a 28% decrease in the total imaging volume (including X-ray, mammography, CT, MRI, ultrasound, interventional radiology, and nuclear medicine) in a 7-week period after the outbreak. The greatest decrease was observed in the 16th week of 2020 for outpatient imaging (88%), affecting all types of procedures including mammography (94%), nuclear medicine (85%), MRI (74%), ultrasound (64%), interventional radiology (56%), CT (46%), and X-ray (22%). The reasons for this decrease were mandatory quarantines and implementation of policies such as the cessation of elective and routine medical services, especially surgical procedures and imaging examinations. Additionally, people's hesitation to use the services due to their fear of being exposed to the Covid-19 virus also contributed to the decrease [34]. Alelyani et al. in the southern Saudi Arabia indicated that the total imaging volume decreased by 22.7% in 2020 compared to 2019. Significant reductions were also observed in April to June (about 42.9%) and July to September (about 44.4%). Regarding departments, the greatest decrease from April to June was observed in outpatient wards (76%) followed by emergency rooms (25%), and the lowest impact was observed in inpatient wards with a decrease of only 6.8%. Concerning the type of procedure, the greatest reductions were reported in nuclear medicine (100%), ultrasound (76%), MRI (74%), and mammography (66%). It seemed that one of the reasons for this decrease was the announcement of the complete closure in the country by the government [33]. Schwertner et al. in Colorado indicated that imaging services decreased significantly during the first wave of the Covid-19 pandemic in March and April 2020, with the greatest decrease in outpatient examinations, reaching 38.7% pre-pandemic level. The decrease in the imaging volume during the first wave of the pandemic could be largely attributed to two factors, including patients’ reluctance to seek medical attention due to the risk of infection and deliberate postponement of non-emergency examinations to create social distancing from patients and staff and restrict the spread of the virus [35]. Parikh in Ohio reported a 55% reduction in the use of imaging services over an 8-week period from the outbreak of Covid-19 in hospitals, and indicated that outpatient examinations, emergency services, and inpatient examinations decreased by 68%, 48%, and 31%, respectively. Mammography and nuclear medicine scans were most affected by the prevalence of Covid-19 with a reduction of 93% and 61%, respectively, the reasons for which were said to be demographic differences and policy changes [36]. Siegal et al. found the drastic reduction in the volume of radiological procedures was challenging, especially in areas most severely affected by the virus. In some areas, reductions in radiological services volume of over 65% were reported. A hospital in New York, for example, experienced an 80% reduction in the total volume of radiology services [37].

Based on the results, the Covid-19 pandemic led to a reduction in drug utilization in the private sector in the short term, but no difference was observed in the long term. In the public sector, however, medicine consumption increased in the short run but did not change in the long term. The decrease in medicine consumption in the short term appeared to be due to the lack of patient referrals resulting from the fear of Covid-19 infection and non-follow-up of non- Covid-19 diseases. On the other hand, the increase in medicine utilization in the short term in the public sector, compared to the private sector, could be due to the referrals of Covid-19 patients and people seeking necessary healthcare services and medication in the public centers. According to the WHO, prevention and treatment of non-communicable diseases have been severely disrupted since the onset of the Covid-19 pandemic. Many people have not received medication for cancer, cardiovascular disease, or diabetes since the onset of the pandemic [8]. As the pandemic continues to spread, medical centers have been focusing on Covid-19 patients, and as a result, chronic patients have not been able to obtain necessary referrals to their specialists. Consequently, they have had to rely on their old prescriptions to buy medicine or consult with pharmacists until being re-examined by physicians [38]. It has been noted that during the pandemic, the spread of unreliable and distorted information has led to panic buying behavior among people, including an increased demand for certain drugs such as chloroquine, hydroxychloroquine, nonsteroidal anti-inflammatory drugs, angiotensin-converting enzyme inhibitors, angiotensin II receptor antagonists, Favipiravir, and Umifenovir. This has led to the coining of the term 'infodemic' to describe the dissemination of such unreliable information [39]. This could be a justification for increased drug consumption during the pandemic in Iran.

The findings showed that the Covid-19 pandemic reduced the utilization of private inpatient services in the short term, but no difference was observed in the long run. Additionally, there was no difference between the public and private sectors in the short and long terms. The decrease in hospitalization at the beginning of the pandemic outbreak might be due to the people's fear of seeking medical attention, and this could be addressed in the public sector by the referring Covid-19 patients to hospitals [40, 41]. Diegoli et al. in Brazil reported a decline in the admission rate of the patients with Ischemic Attack from an average of 12.9% in 2019 to 8.3% after the pandemic. The reason for decreased referrals in their study was people’s fear of going to the hospital due to the infection and the confusion regarding the lockdown protocols. Another possibility was that social constraints caused mild symptoms of the disease to be neglected. In particular, reducing industrial activities and vehicle traffic and creating social constraints could be effective in reducing the incidence of some diseases [41]. In their study, Birkmeyer et al. showed a significant decline in hospital admissions in the United States with the onset of the Covid-19 pandemic in 2019. Even in hospitals that experienced the least impacts from Covid-19 admissions, non-Covid-19 admissions dropped by 39.5%. The hospitals with the most Covid-19 effects experienced a decline of 50% in non-Covid-19 admissions. According to this study, people's fear of getting infected by the disease decreased hospital admissions and increased the use of home care services [40]. Nourazari et al. in Massachusetts reported that emergency room services decreased significantly during the Covid-19 pandemic. There was a relative change in hospital admissions through emergency centers during the pandemic. Significant reductions in admissions were observed in the children, women, Medicare patients, and a variety of diseases related to chronic respiratory conditions and behavioral health. Patients who required hospitalization accounted for a significant proportion of the reductions. This suggests that many patients postponed necessary medical care. The decline in hospital admissions during the pandemic, was a reflection of several factors. The first one, which directly affected admissions, could be explained by striking balance between the urgency of an emergent health need and the concern about the risk of Covid-19 infection. It might also be due to reduced travels by car (resulting in fewer car accidents), improved air quality, and reduced transmission of infectious diseases due to social distancing and the use of masks. There is also evidence of better drug adaptation for chronic diseases, and greater awareness of maintaining health during the pandemic [42]. Oseran et al. compared the billing data of eight acute care hospitals and found a 33.7% reduction in admission rates one year before and one year after the Covid-19 pandemic [43]. According to a study by Filippo et al., the admission rate of Acute Coronary Syndrome (ACS) patients in 15 hospitals in northern Italy was 13.3% per day during the study period (February 20 to March 31, 2020), which was significantly lower than the corresponding rate in the two control periods (18%, 18.9%). Additionally, a further decrease in the admission rate was observed after the implementation of quarantine measures [13].

The Covid-19 pandemic had a significant impact on people's lifestyles, leading to changes in work habits, social interactions, and leisure activities. The restrictions imposed during the Covid-19 pandemic, including social distancing measures and lockdowns, had a significant impact on the utilization of healthcare routine services.

According to the current study, there was a decline in referrals to private and public healthcare providers for major health services in the early stages of the pandemic, as individuals were reluctant to seek care for non-Covid-19 conditions due to fears of exposure to the virus. Overall, the results indicated that the utilization of services (including laboratory, radiology, medication, and hospitalization) in private centers decreased in the short term, but either increased or remained unchanged in the long term. Compared to the private sector, radiology services decreased in the public sector in the short term but medication increased. Other services did not change, though. In the long run, the utilization of laboratory services decreased, while other services remained unchanged.

The decrease in the utilization of private healthcare services was more pronounced, as these services are typically elective or luxury in nature. However, as the pandemic continued and chronic and non-Covid-19 cases went untreated, some illnesses worsened, leading to a return to normal levels of healthcare consumption. With the advent of vaccinations and increased community awareness about living with the pandemic, people have gradually returned to their pre-pandemic lifestyle and resumed seeking treatment for chronic and non-Covid-19 illnesses.

Despite a decline in overall healthcare utilization during the pandemic, there was an initial increase in the use of inpatient services and medication in the public sector. This was likely due to the admission and treatment of Covid-19 patients in public hospitals, as well as a shift of patients from private to public healthcare providers. Furthermore, emergency services, traumas, and other essential services for which the patients had to refer to receive treatment and medication did not change or increase inpatient and drug services in the public sector compared to the private one.

Similar to other studies, our study also has some limitations, including a small number of time points (sample size in time series analysis), failure to check and address the seasonality of the data due to the limited number of time points, and surveying only the insured individuals of a single insurance organization, even though this insurer covers over 50% of the population in Fars province.

Like many other studies, our research has several limitations. One of these limitations was the small number of time points in the study, which indicates the sample size in time series analysis. Additionally, due to the limited number of time points, we were unable to account for any seasonality in the data. Furthermore, our study only surveyed insured individuals from a single insurance organization, even though this insurer covers over 50% of the population in Fars province. Despite the mentioned limitations, the present study is one of the few studies that has estimated the impact of Covid-19 on healthcare service utilization by public and private sectors, using MGITSA. Furthermore, we assessed the impact of Covid-19 on various services, including laboratory, medication, and radiology services.

## Conclusions

Utilization of health services in the public and private sectors, except for medication and hospitalization, decreased significantly in the short term, but most services returned to the previous status in the long run. The decrease in access to health services, at least in the short term, can impose a significant burden of various diseases and increase health costs in the coming years. Considering the changes in service utilization in pandemics, it seems necessary for policymakers to appropriately and promptly respond to crises in order to prevent disruption in the process of access to health services, especially emergency services for non-communicable diseases.

In order to increasing rational health care utilization in both private and especially public sectors after pandemics, increasing services delivery capacity such as bed density or work force activity time, improving financial protection by insurance especially for private sector and developing a system to follow up patients with chronic diseases are proposed.

## Data Availability

The datasets gathered and analyzed during the current study are available from the corresponding author on reasonable request.

## Abbreviations

ACS: Acute Coronary Syndrome
ITSA: Interrupted Time Series Analysis
MGITSA: Multiple Group Interrupted Time Series Analysis
NRLs: National tuberculosis Reference Laboratories
WHO: World Health Organization
